# Variations in regulations to control standards for training and licensing of physicians: a multi-country comparison

**DOI:** 10.1186/s12960-021-00629-5

**Published:** 2021-07-23

**Authors:** Wafa Aftab, Mishal Khan, Sonia Rego, Nishant Chavan, Afifah Rahman-Shepherd, Isha Sharma, Shishi Wu, Zahra Zeinali, Rumina Hasan, Sameen Siddiqi

**Affiliations:** 1 Aga Khan University, Karachi, Pakistan; 2 London School of Hygiene and Tropical Medicine, Keppel St, Bloomsbury, London, WC1E 7HT United Kingdom; 3 Independent Consultant, Mumbai, India; 4 Independent Consultant, Delhi, India; 5 National University of Singapore, Singapore, Singapore; 6 Independent Consultant, Tehran, Iran

**Keywords:** Governance, Medical training, Human resources

## Abstract

**Background:**

To strengthen health systems, the shortage of physicians globally needs to be addressed. However, efforts to increase the numbers of physicians must be balanced with controls on medical education imparted and the professionalism of doctors licensed to practise medicine.

**Methods:**

We conducted a multi-country comparison of mandatory regulations and voluntary guidelines to control standards for medical education, clinical training, licensing and re-licensing of doctors. We purposively selected seven case-study countries with differing health systems and income levels: Canada, China, India, Iran, Pakistan, UK and USA. Using an analytical framework to assess regulations at four sequential stages of the medical education to relicensing pathway, we extracted information from: systematically collected scientific and grey literature and online news articles, websites of regulatory bodies in study countries, and standardised input from researchers and medical professionals familiar with rules in the study countries.

**Results:**

The strictest controls we identified to reduce variations in medical training, licensing and re-licensing of doctors between different medical colleges, and across different regions within a country, include: medical education delivery restricted to public sector institutions; uniform, national examinations for medical college admission and licensing; and standardised national requirements for relicensing linked to demonstration of competence. However, countries analysed used different combinations of controls, balancing the strictness of controls across the four stages.

**Conclusions:**

While there is no gold standard model for medical education and practise regulation, examining the combinations of controls used in different countries enables identification of innovations and regulatory approaches to address specific contextual challenges, such as decentralisation of regulations to sub-national bodies or privatisation of medical education. Looking at the full continuum from medical education to licensing is valuable to understand how countries balance the strictness of controls at different stages. Further research is needed to understand how regulating authorities, policy-makers and medical associations can find the right balance of standardisation and context-based flexibility to produce well-rounded physicians.

## Introduction

Addressing the shortage of healthcare providers—which is essential for improving health worldwide—poses a conundrum for health policy-makers. There is an urgent need to train more healthcare providers to address the estimated needs-based shortfall of 17.4 million globally [1]. At the same time, there is a growing body of literature raising concerns about insufficient attention to minimum standards of professional education and licensing, and the professionalism of healthcare providers as a result [2–5]. Whilst the number of medical doctors in the health workforce is growing globally by more than 400,000 every year, variation in the level of professionalism—which includes technical competence, clinical skills and ethical conduct—is huge [3, 4, 6]. Serious systemic issues underlying this variation are illustrated by reports of the explosive growth of for-profit medical colleges with low teaching and examination standards, impersonation fraud used in medical college entrance exams, and bribery as a route into some medical colleges [7–12].

In this paper, we focus on regulation across the full professional development continuum from medical education and clinical training, to physician licensing and re-licensing. We examine the types of controls in place to ensure a minimum standard of professionalism among licensed doctors, recognising that controls along this continuum may influence the ultimate quality of care that they provide [13]. There is clear evidence that insufficient control on standards of medical education, training and licensing warrants attention as it allows large variations in class sizes, examination procedures, core curricula and basic clinical competence within the same country [14–17]. Yet there is a gap in the literature, particularly with respect to studies that consider the intersection of medical education and workforce regulation policy [13, 18]; most studies look at specific processes in isolation, such as licensing or continuing medical education (CME) [19, 20], rather than the combination of controls applied across the professional development continuum.

### Analytical framework and objectives

**Fig. 1:**
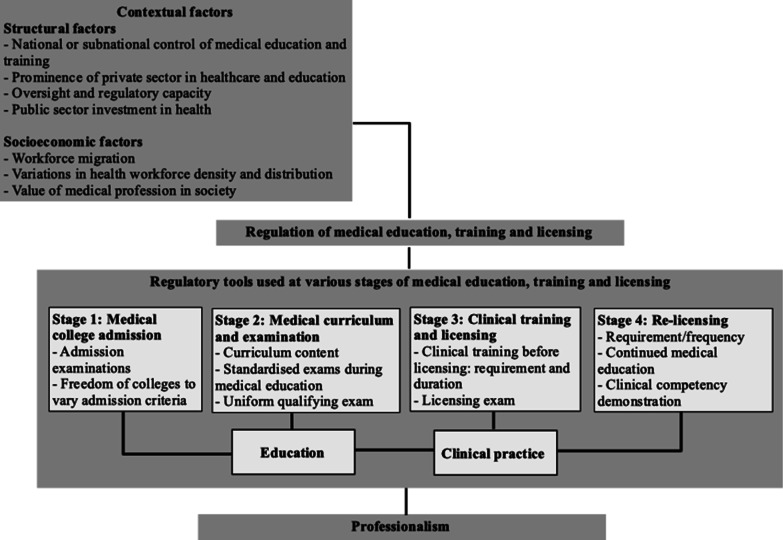
Analytical framework

We developed the analytical framework presented in Fig. 1 to guide our analysis. It outlines four stages—from admission in medical college to licensing and re-licensing—at which regulatory controls can act. It also emphasises that contextual factors can influence the type of regulatory approach. The framework was designed based on a review of seminal literature on the quality of medical care and medical education [4, 5, 21, 22], and the division of medical education components proposed by Zhu et al. [23]. The four stages reflect factors affecting the quality of the health workforce as described by Burdick and Dhillon [13], including who is chosen to enter the profession (Stage 1), how and what they are taught (Stage 2), how they are determined to be qualified (Stages 2 and 3), and how they maintain and update their skills (Stage 4).

Since terminology varies across countries, we refer to medical degree administering institutions as ‘medical colleges’ and to the period of clinical training between graduation from medical college and licensing as ‘clinical training’.

The literature indicates that there is a multiplicity of regulatory tools and approaches available at each of the four stages, depending on the country and/or regional context. Studies of regulatory regimes repeatedly refer to a mix of approaches, with little consistency across different country contexts [19, 24, 25], and there is no agreement on which regulatory approaches might be most effective in ensuring professionalism. Indeed, no model emerges as ‘gold standard’ for regulation that is applicable across diverse contexts [19, 25]. For instance, there is no ideal approach to determining the frequency of re-licensing (stage 4 on our framework) and the modalities and frequency of CME. The most commonly used standards, from the World Federation of Medical Education, cover basic medical education, post-graduate medical education and continuing professional development [26]. The standards do not cover licensing, nor are they prescriptive. As stated by the World Federation of Medical Education, their standards are intended to provide a framework for educational institutions and accrediting agencies to develop and evaluate medical education as appropriate for the context [27].

In light of the lack of research looking at the entire continuum from medical education and training to licensing and re-licensing, and comparing approaches used across diverse contexts, our study examines how different national and sub-national agencies apply the various available regulatory controls to different stages in this continuum. We discuss possible implications of different regulatory approaches applied and seek to identify innovations in models of regulation that could be useful to learn from.

## Methods

In line with our analytical framework, we purposively selected seven countries from North America, Europe and Asia. We limited our scope to geographical regions where the research team had expertise and connections owing to the need for extensive validation of information by local experts. Within these regions, we selected countries that differed on key attributes that could underpin variations in the types and quality of regulations. These attributes included: (i) gross national income (as classified by the World Bank) [28]; (ii) whether regulations relating to medical education and licensing were controlled by national or sub-national authorities; and (iii) extent of private sector presence in medical education (Table 1).

**Table 1:** Overview of case-study countries [74, 75]

Indicator	Country						
	Canada	China	India	Iran	Pakistan	UK	USA
World Bank income classification	High	Upper middle	Lower middle	Upper middle	Lower middle	High	High
Population size (2019)	38 million	1.4 billion	1.3 billion	83 million	217 million	67 million	328 million
General government expenditure on health as a percentage of total government expenditure (2018)	20%	9%	3%	22%	5%	19%	23%
Physicians per 1000 population (2018/19)	2.8	2.0	0.8	1.6	1.0	3.0	2.6
Regulation of medical education and licencing led by national authorities or sub-national authorities?	Sub-national	Sub-national	Sub-national	National	Licencing by national body; medical education by sub-national	National	Sub-national

Focusing on regulations and guidelines that relate to the four stages in our analytical framework within each country, we collected information from three sources: websites of relevant regulating bodies in each country (such as national medical councils), a systematic search for scientific and grey literature and news articles published online, and written input from 1–2 medically qualified researchers or medical professionals with lived experience and knowledge of each of following countries: China, India, Iran, and Pakistan. For the latter, we used standardised questions that solicited information about each of the stages in our analytical framework. Our systematic search methodology is summarised in the appendix (Appendix 1). Information from these three sources was analysed by following three steps. First, we extracted relevant information about each country using an MS Excel-based template with sections mirroring our analytical framework. Second, we examined the standardised information collected about processes and regulations in each study country individually, from stages one to four in the framework. Third, we summarised the information at key steps into either yes/no answers for the presence of a regulation, or into years (of study), to allow cross-country comparisons.

## Results

Table 2 presents a summary of our comparative analysis, and below we synthesise the key findings using subheadings that correspond to the stages in our analytical framework. Some overarching differences are summarised here.

**Table 2:** Comparison of regulations for medical education, clinical training, licensing and re-licensing across the four stages of the analytical framework and across the case-study countries

Stage	Regulations	Can	China	India	Iran	Pak	UK	USA
N/A	Are private (for-profit) medical colleges allowed?	No	Yes	Yes	Yes	Yes	No	Yes
1	Is there a uniform examination that all students undertake for admission to medical college?	Yes	Yes	Yes	Yes	Yes	Yes	Yes
2	Is there a uniform examination for acquiring a medical degree?	Yes	Yes	No	Yes	No	No	Yes
2	Is medical training offered at the undergraduate level/ without first degree?	No	Yes	Yes	Yes	Yes	Yes	No
2,3	Total years of medical education and clinical training (after school) to be eligible for medical licence	9–10	8	5.5	7	6	7	11
3	Is there a national licensing exam?	Yes	Yes	No^a^	No	No^a^	No	Yes
4	Is license renewal by an independent body required across the country	Yes	No^b^	No	Yes	Yes	Yes	Yes
4	After how many years is licensing renewal required, if at all?	1	N/A	5^c^	5	2	5	1–4
4	Does permission to continue medical practise require CME or demonstration of clinical competencies?	Yes	Yes^b^	Yes^c^	Yes	No	Yes	Yes, except in 1 state

^a^Introduced in 2021 (Pakistan) and 2022 (India)

^b^Physicians have to pass a competency test every two years to continue to practise even though licence renewal is not required

^c^Only applies to nine states that have mandatory relicensing

First, we identify large variation in the (usual) number of years of education between completing secondary schooling and being licensed to practise medicine ranging from five and a half years in India to 11 years in the USA. Part of this difference relates to the usual requirement in the USA and Canada to complete a 3 to 4-year undergraduate degree before entering into medical college. Additional training for specialisation varies according to the field and is outside the scope of our analysis. Apart from the UK and Canada, all other countries we analysed allow medical education to be delivered by both public and private medical colleges, although the dominance of private medical education and approaches used to regulate private medical education vary. In Iran, private medical education is typically nested within some of the highest ranking and well-resourced public universities, where students who can afford the fees can receive medical education despite having lower examination scores [29]. These medical colleges also offer education to overseas students, comprised of an initial component in English for the first two to three years, followed by clinical training in Farsi alongside the rest of medical students. In both Pakistan and India, there are now substantially more private than public medical colleges [30, 31], and the medical education and licensing regulators are currently undergoing reforms, after decades. India has introduced the 2019 National Medical Commission (NMC) Bill, which is in the process of implementation, and in Pakistan, a new regulating authority—the Pakistan Medical Commission (PMC) (has been established [32, 33].

### Stage 1: medical college admission

For the countries we analysed, standards for entry into medical education are regulated in three ways: through identical entrance tests to all public and private medical colleges administered by external agencies (USA, UK, and Canada) (Box 1); through standardised tests which are not specific to medical colleges but are used by all undergraduate colleges across the country as part of the admissions decisions, whether public or private (China and Iran); and through admission tests which are not standardised across the country and are instead determined by the state/province (in the case of public colleges) or the medical college itself (in the case of private colleges) (in Pakistan and India prior to the ongoing reforms).

Even when there are stronger controls, through identical or standardised tests, it is often the case that the top-tier medical colleges will only accept students with high testing scores, whilst some private medical colleges will accept students with weaker scores. For example, when it comes to overseas students, the entry requirements for medical colleges in Iran vary depending on the college and are typically less rigorous. Similarly, in China, admission decisions for overseas students are managed by the college. Changes to medical college admissions rules are highly political. In Pakistan, while a uniform admission test is necessary to enter all private and public medical colleges from 2021 onwards, there are ongoing disputes about whether private colleges are at liberty to decide what weighting to give to the entrance exam results in admission decisions [34]. To centralise and standardise admissions procedures nationwide, the Indian union government introduced the National Eligibility Cum Entrance Test for admission into all medical and dental programmes in 2012. This was ruled unconstitutional from 2013 to 2016 and is now reinstated as the sole admission criterion under the NMC Bill 2019 [35].

Box 1: Medical college admissions *Examination specific for students applying to medical colleges*

Canada—Medical College Admissions Test (except Quebec).

India—National Eligibility Entrance Test.

Pakistan—Medical and Dental College Admissions Test.

UK—UK Clinical Aptitude Test or BioMedical Admissions Test.

USA—Medical College Admissions Test.

*Examination for all undergraduate education*

China—National College Entrance Examination (Gaokao).

Iran—Iranian University Entrance Exam (Konkour).

### Stage 2: medical curriculum and examination

The USA, UK, Iran and Canada have standardised medical education curricula and qualifying examinations for receiving a medical degree that are overseen by third-party governing bodies, enabling relatively strict controls over standards for graduating doctors [36–41]. The USA, Iran and Canada have multi-part examinations which are identical for all students in all medical colleges. In Iran, all medical students are required to take uniform, standardised examinations twice during medical college: basic sciences in the third year and a clinical knowledge assessment in the fifth or sixth year. All students additionally take a clinical competency examination focusing on practical skills in their final year prior to receiving the diploma. The UK has weaker controls at the examination stage; medical examinations are reviewed to meet assessment standards, but vary from college to college [42]. However, a new examination for all UK medical colleges, the Medical Licensing Assessment, will begin in 2024 [43].

In China, the curricula covered and teaching materials used may vary slightly between medical colleges, depending on additional teaching materials colleges select to complement standardised textbooks, although the Ministry of Education and Ministry of Health have clear guidelines on this [44]. Although qualifying examinations administered by each medical college can differ, the National Medical Licensing Examination is the same across the country.

In Pakistan, there are guidelines on the minimum content required in each of the compulsory subjects in medical colleges, but no enforced regulation. Pakistan has no standardised examination to receive a medical degree, although a National Licensing Exam has been introduced which is planned to be implemented from 2021. Under the NMC Bill 2019, India is shifting to a competency based undergraduate curriculum that applies to public and private medical colleges, and is overseen by the newly introduced NMC [45]. It is also introducing a two-part National Exit Test, which should be in place by 2022, and will be a requirement for entry into postgraduate training and registration in the state and national registers [46]. In recent years, however, qualifying examinations have varied across colleges in Pakistan and India, such that it can be easier to receive a nationally recognised medical degree from some colleges.

### Stage 3: clinical training and licensing

A medical licence permits a person to legally practise medicine, and all countries have a method of licensing medical doctors following completion of mandatory clinical training. The latter typically involves the graduate doctor conducting clinical work under the supervision of a licensed clinician.

The USA and China have the longest durations of clinical training (3 years) which must be completed in a government approved facility. Following clinical training in the USA, a licence is granted after completing the three-step licensing exam [3]. China’s standardised residency training, which has been mandatory from 2020, is divided into two-and-a-half years of hospital-based training and 6 months in a community healthcare facility [47]. This is followed by an assessment to obtain a certificate of completion of residency training, which is recognised nationwide [48]. Doctors need two certificates to be licensed to practise medicine in China. To receive the Medical Practitioner's Qualification Certificate, graduates must pass the National Medical Licensing Examination and complete postgraduate training. The Physician’s Practice License is obtained after joining a specific medical facility and needs to be re-applied for if the doctor moves to another place of work.

The UK and Canada require two years of clinical training in government-funded and accredited hospitals. Proof of completion of two years of clinical training is required for licensing, and further specialty training is often undertaken [43, 49].

In Iran, there are some differences depending on whether the student intends to practise inside or outside of Iran. All students go through 18 months of clinical training (internship) prior to official graduation. In their role as interns, they provide essential services at the Ministry of Health-affiliated teaching hospitals under the supervision of licensed physicians and residents. After completing medical college and the clinical training period, a licence to practise is issued following a paid mandatory government service provided to areas with a shortage of physicians, mainly rural or low-resource areas, taking between 14 to 24 months [50]. Overseas students are not required to do the mandatory rural area service post-graduation if they intend to practise outside of Iran.

Pakistan and India have the shortest clinical training periods of 12 months. Such training is mandatory for receiving a licence and this must be based in a recognised teaching institution. In India, six months are reserved for rural or community health facilities [51, 52]. Mechanisms to check and enforce minimum standards of supervision and clinical training in teaching hospitals, such as accreditation or audits by external agencies, are not used uniformly yet.

### Stage 4: re-licensing

Rules on medical licence renewal and CME across the seven countries can be broadly categorised as: mandatory licence renewal tied to CME or demonstration of some competencies (most states in USA, Canada, UK, and Iran), licence renewal mandatory but only requires payment of a fee under the PMDC rules (Pakistan) and no requirement for licence renewal by an independent body (China and majority of states in India) [40, 53, 54]. Although there is no expiration date for the Medical Practitioner’s qualification certificate in China, CME is compulsory, as is passing a performance assessment supervised by the local health departments every 2 years; doctors who fail are suspended from practising for 3–6 months until they pass the assessment [55]. There are also assessments conducted by government health facilities, which means that CME is tied to the doctors' career progression, but variation in assessment standards is possible [20]. Whilst CME completion for doctors working in government health facilities is high, the enforcement of CME regulations and performance assessments on the relatively small proportion of doctors working solely in private clinics is not clear [47].

In the USA, whilst all states require re-licensing, the specific requirements for renewal varies by state. Only one state (Montana) has no CME requirement for medical doctors to maintain their medical licences [56, 57]. Although the Medical Council of India made a mandatory resolution in 2011 requiring doctors to attend minimum of 30 h of CME every five years to ensure re-registration, only nine of 26 State Medical Councils have made re-registration for licence renewal mandatory [58]; therefore only about 20% of India's doctors follow CME rules, as they are not legally required in the states in which they work [20, 59]. In Pakistan, registration needs to be renewed every two years; licence renewal does not, however, require any additional assessments or CME, only payment of a fee, but this may change under the new PMC [14].

## Discussion

Our analysis shows that there are wide variations in the controls that are employed by countries to regulate medical education, clinical training and physician licensing and re-licensing, and that there is value in analysing the entire continuum, as we do in this study, because strong controls at one stage are often balanced with less strict controls at other stages. Based on our findings and in line with what others have reported, we would propose it is not practical or desirable to provide a gold standard of regulatory approaches to be applied across different contexts, and that the strictest controls may not always be appropriate [19, 25, 60]. In addition, conceptualising regulatory controls as operating on a continuum—from medical college admission to relicensing and continued professional development—rather than as discrete controls independent of checks at other stages, opens up a broader set of regulatory options and offers more flexibility based on what is required or feasible at different stages. For example, if there is a strongly enforced minimum standard for medical school graduates, this may reduce the need for an additional nationally standardised examination at the admission to medical college stage.

Indeed, the development of a gold standard for regulation of medical education, clinical training, licensing and relicensing risks producing a one-size-fits-all approach that is excessively reliant on Western approaches [61]. We therefore focused instead on drawing lessons through a comparison of models used by different countries, highlighting specific considerations about controls that can be used at each stage of our analytical framework, and discussing how contextual factors might influence decisions on appropriate controls to use.

Considering the first stage of our framework, medical college admission, we note that although having uniform admission examinations across all colleges is a control often used to standardise the competencies of admitted candidates, allowing colleges the freedom to select candidates without a mandatory minimum score dilutes the effect of this attempt at standardisation resulting in variable enrolment standards even in countries with uniform admission examinations. For example, a study from China showed that private medical colleges often require lower scores than public colleges [62]. An advantage of having uniform examinations linked to a minimum score for admission is that it prevents profit-making colleges from lowering standards to allow fee-paying students without sufficient competence to be admitted. A uniform national standard of competence required at the graduation and/or licensing stage can also help to ensure that variations in admission standards do not adversely impact levels of professionalism among practising doctors. However, there may be value in some medical schools allowing lower admissions scores in contexts where there is large sub-national variation in education standards or under-representation of specific groups in the medical profession. Less emphasis on examination scores in admissions decisions may also reflect a growing realisation that other skills, such as strong communication and ability to collaborate, is worth considering in addition to academic achievements [63, 64]. Alternatively, minimum admissions standards for medical colleges may be an incentive for the providers of primary and secondary education in less-developed regions to improve the quality of education.

In relation to stages two and three in our framework, we found that all three countries (UK, Pakistan and India) that do not have uniform licensing examinations are currently in the processes of introducing these as a tool to control variations in standards. In Canada, where physicians are licensed by provincial authorities to practise in their jurisdiction, calls have been made to create a national license for physicians to standardise licensing across provinces so that it is easier to redistribute physician workforce and reduce gaps in physician coverage [65]. Clinical training in a teaching hospital is mandatory prior to licensing in all countries, but the quality of this training depends on the training institution within which graduate doctors are embedded and the supervision received; this is difficult to monitor and subject to variable levels of controls. For example, there is evidence of private medical colleges that are not linked to appropriate teaching hospitals and clinical laboratories compromising the quality of mandatory clinical training [66]. As the number of medical colleges grow in a country, it is important to ensure that each one is linked to an appropriate teaching hospital where graduates can receive high quality clinical training. Regulations on the minimum years that an accredited teaching hospital has been running for, and on essential medical departments that teaching hospitals must have, may be useful here [67].

With respect to the final re-licensing stage of our framework, we found that substantial variations in re-licensing requirements across a country occurred when sub-national rather than national authorities are responsible for setting rules (for example, in India and USA). In the USA, the states of Indiana and New York only require “opioid prescribing and opioid abuse” training as part of their mandatory CME, and Montana does not require any CME for re-licensing, but all others do. We also found that licensing can be de-linked from CME requirements or demonstration of knowledge or skills (in Pakistan and Montana state, in the USA). Both of these situations make it possible for doctors to legally practise for decades after initial licensing, without any check on their continuing clinical competence. In another model de-linking re-licensing and competence, in China, re-licensing is not required but physicians need to demonstrate adequate competency in an exam every two years to continue to practise. An unusually lax model exists in all except nine states of India [68], where physicians do not require any re-licensing by an independent regulator to continue to practise after acquiring the initial licence. Whether physician competencies can be reliably maintained in systems without mandatory assessments to allow continued medical practise, and whether controls at other points help to maintain professional standards is a relevant question. Presence of such highly variable approaches to relicensing within sub-national entities of the same country, such as in India and the US, highlights the fact that there is little evidence on the effectiveness of various models in maintaining physician competencies and to guide policy choices. Such divergent models operating in the same country provide an opportunity to study their comparative effectiveness and strengths or weaknesses as regulatory models to maintain physician competence.

Although inadequate controls on the quality of medical education and clinical training is a challenge to address in both public and private sector institutions, the profit-making business model of private medical colleges introduces specific complexities with respect to regulation [4]. A comparison across our case-study countries was insightful here as we could identify three approaches to regulating (private) medical education: retaining medical education in the public sector (UK, Canada); having uniform minimum entry criteria and licensing examinations that apply to public and private colleges (China and USA); and an innovative approach of embedding private sector colleges within public universities (Iran). Pakistan and India are currently changing their regulations, aiming to move towards the second approach.

While investing in public sector medical education and retaining all or most medical education in the public sector has many advantages, and can reduce the layers of controls needed, private medical education is often encouraged because it allows injection of private capital into medical education to increase the number of doctors without government investment [4, 69]. The USA shows that relatively strong controls on medical education are possible despite having a large and powerful private medical education sector. In countries where regulatory authorities have not strengthened at the pace at which the private medical education sector has grown, however, challenges in regulation can occur. In India and Pakistan, private medical colleges have mushroomed in the last two decades, but with increasingly lax regulations because the rapid private medical college expansion has outstripped the capacity of regulatory systems. It is important for countries to be cognisant of becoming trapped in a cycle in which weak regulations on minimum standards for faculty and infrastructure, and on profit-making, allows the number of highly profitable medical colleges to grow rapidly, and potentially enables the powerful private sector to engage in lobbying or co-opting the regulatory process to weaken regulatory controls [70]. Suggested approaches include creation of dedicated departments of medical education in private colleges, relying on system of accreditation and enhancing the capacity and powers of regulatory bodies [15, 71].

Although our comparative analysis of regulations and guidelines yielded some important insights, we acknowledge limitations of our study scope; for example, we do not consider rules relating to foreign medical graduates or compare teaching methods. Another limitation is that we focused on regulations as written (de jure), regardless of whether the practice is implemented in reality (de facto). Further study on the strength of regulatory bodies in different countries, involving primary qualitative research with regulators, and on the relationship between regulatory controls at various stages of medical education and clinical competence assurance of practising doctors would be useful.

## Conclusions

Doctors typically hold a position of power and are trusted by patients because of the uncertainty, informational asymmetry, and buyer vulnerability that characterise medical consultations [72, 73]. The professionalism of doctors, and the measures put in place by relevant authorities to ensure their professionalism, are, therefore, paramount. While there is no gold standard model for medical education and practise regulation—even among high-income countries that are perceived as leaders in this area—examining the combinations of controls used in different countries enables identification of innovations and of regulatory approaches that have been used to address specific contextual challenges, such as decentralisation of regulations to sub-national bodies or privatisation of medical education. Our study highlights the value in looking across the full continuum of professional development from who is allowed to enter the medical education, what students are taught, how they are determined to be qualified to practise medicine and how they must maintain and update their skills; using our analytical framework to examine the full continuum showed that countries may balance having weaker controls at one stage with stricter controls at an earlier or later stage. Finally, whilst we identified a shift towards introduction of stronger regulatory controls, such as a national licensing examination in countries that do not have this yet, we emphasise that further work is needed to understand how regulating authorities, policy-makers and medical associations can find the right balance of standardisation and context-based flexibility, and between students’ theoretical, and ethical and practical skills.

## Data Availability

All data generated or analysed during this study are included in this published article.

## Appendix 1

See Table 3.

**Table 3:** Systematic search strategy

Search platform	Google (UK)
Search date	15 September 2020
Search terms	Country name + Regulation* + one of the terms below:
	Medical Education
	Medical education entrance exam*
	Medical education curriculum
	Medical education exam*
	Medical degree exam*
	Medical license*
	Medical postgraduate training
	Continuing medical education
Review process	Review first 10 results for each search combination [56 searches in total]

## Abbreviations

CME: Continued medical education
NMC: National Medical Commission
PMC: Pakistan Medical Commission
SRT: Standardised residency training
